# Investigating the effect of reduced temperatures on the efficacy of rhabdovirus-based viral vector platforms

**DOI:** 10.1099/jgv.0.002010

**Published:** 2024-08-22

**Authors:** Julia E. Kakish, Yeganeh Mehrani, Arthane Kodeeswaran, Katrina Geronimo, Mary Ellen Clark, Jacob P. van Vloten, Khalil Karimi, Bonnie A. Mallard, Baozhong Meng, Byram W. Bridle, Jason P. Knapp

**Affiliations:** 1 Department of Pathobiology, University of Guelph, Guelph, Ontario, N1G 2W1, Canada; 2 Department of Molecular and Cellular Biology, University of Guelph, Guelph, Ontario, N1G 2W1, Canada

**Keywords:** oncolytic, rhabdovirus, temperature, viral vector

## Abstract

Rhabdoviral vectors can induce lysis of cancer cells. While studied almost exclusively at 37 °C, viruses are subject to a range of temperatures *in vivo*, including temperatures ≤31 °C. Despite potential implications, the effect of temperatures <37 °C on the performance of rhabdoviral vectors is unknown. We investigated the effect of low anatomical temperatures on two rhabdoviruses, vesicular stomatitis virus (VSV) and Maraba virus (MG1). Using a metabolic resazurin assay, VSV- and MG1-mediated oncolysis was characterized in a panel of cell lines at 28, 31, 34 and 37 °C. The oncolytic ability of both viruses was hindered at 31 and 28 °C. Cold adaptation of both viruses was attempted as a mitigation strategy. Viruses were serially passaged at decreasing temperatures in an attempt to induce mutations. Unfortunately, the cold-adaptation strategies failed to potentiate the oncolytic activity of the viruses at temperatures <37 °C. Interestingly, we discovered that viral replication was unaffected at low temperatures despite the abrogation of oncolytic activity. In contrast, the proliferation of cancer cells was reduced at low temperatures. Equivalent oncolytic effects could be achieved if cells at low temperatures were treated with viruses for longer times. This suggests that rhabdovirus-mediated oncolysis could be compromised at low temperatures *in vivo* where therapeutic windows are limited.

## Data Availability

The datasets used and analysed during the current study are available from the corresponding author on reasonable request.

## Importance

Cancers have a weakness that can be taken advantage of for treatment. They are often impaired in their ability to protect themselves against viruses. Oncolytic viruses do not cause disease in normal tissues but can often replicate out of control and kill cancer cells. Rhabdoviruses have long been studied as stereotypical oncolytic viruses. We report for the first time that oncolytic rhabdoviruses are impaired in their ability to mediate the death of cancer cells at anatomical temperatures <37 °C. When evaluating the mechanisms underlying this barrier and, in our attempt to mitigate it, we found that the problem was with cancer cells, which died more slowly at lower temperatures, whereas the oncolytic virus maintained its ability to replicate. This highlights a unique clinical problem, namely that relatively low anatomical body temperatures may represent a barrier to the oncolytic potential of rhabdoviruses due to inherently short therapeutic windows in immunocompetent hosts.

## Introduction

Cancers are leading causes of death globally. Cancer-associated cases and deaths are expected to continue to rise as populations adopt lifestyle changes that increase cancer risk [1]. Oncolytic virus (OV)-based therapeutics are potential tools for the treatment of cancers. OVs replicate selectively in cancer cells, because during tumorigenesis cancer cells often accumulate mutations in genes involved in antiviral pathways [2]. This allows OVs to preferentially replicate in cancer cells, leading to cell death and the initiation of an anti-tumour immune response.

Optimizing viral vectors could increase their therapeutic abilities when treating diseases at sites of the body that are subject to extreme temperatures. This may include anatomical sites that are below the average body temperature of 37 °C. However, the effect of low temperature on the efficacy of viral vectors has not been extensively researched, despite this having important implications for clinical applications.

Body temperature varies by anatomical location and with environmental variables. Within the respiratory pathway, the temperature of the nasal passages can range from 32 to 34 °C, only reaching 37 °C in the lower lungs, and skin temperature can drop well below 28 °C for peripheral areas such as the hands and feet when in cold ambient conditions [3,5]. This must be considered when administering viral vectors at these sites or if systemically administered vectors traffic to tissues at low temperatures. For example, OVs used to treat head and neck cancers within the upper respiratory tract, melanomas on the skin or testicular cancers would be tasked with functioning at relatively low anatomical temperatures. Several studies have already identified that tumours, including brain tumours, and soft tissue tumours, such as lipomas and atypical lipomatous tumours, can be ~2 °C cooler than surrounding healthy tissues due to multiple contributing factors [6,10]. These may include a low density of tumour microvessels, tumour necrosis and lower metabolism in the area surrounding the tumour [11]. Despite extensive research done on viral vectors, to the best of our knowledge, functional studies of the effectiveness of these viruses at low temperatures are lacking.

To investigate the effect of low temperatures on viral vectors, two stereotypical rhabdoviruses were utilized, vesicular stomatitis virus (VSV) and Maraba virus (MG1). Both of these rhabdoviruses have undergone extensive pre-clinical testing as viral vectors. MG1 and VSV have proven to be efficacious cancer immunotherapies in pre-clinical and translational models and can boost adaptive anti-tumour immunity, such as in the treatment of melanomas [12,14].

These two rhabdoviruses have also undergone testing in clinical trials. VSV has been tested as a viral vector-based vaccine against Ebola virus and shown to initiate an adaptive immune response with only mild adverse effects [15,16]. MG1 has also undergone testing in clinical trials, in combination with an adenoviral vector, to treat melanomas and was shown to replicate in humans and induce anti-tumour immunity [17].

**Fig. 1.**
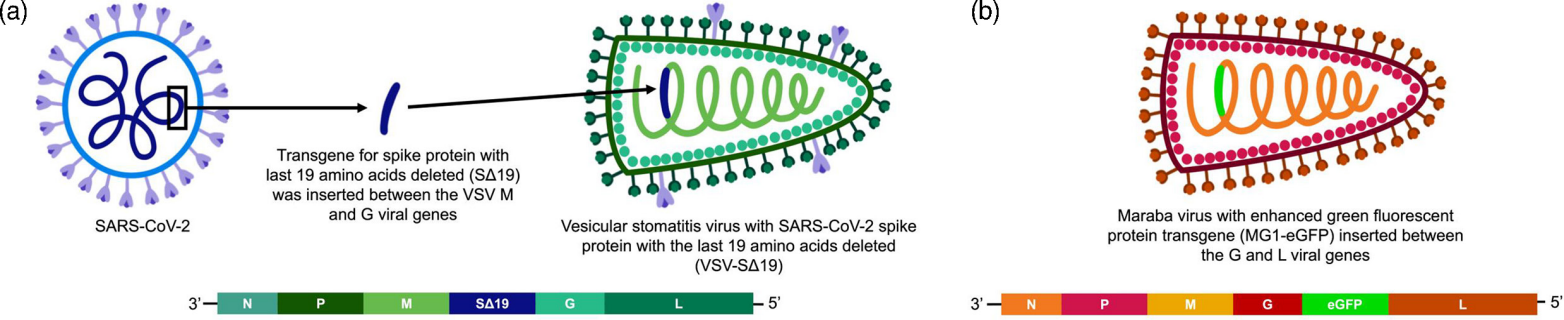
Illustrations of the vesicular stomatitis virus (VSV) and Maraba virus (MG1) vectors. (**a**) The VSV vector had a transgene for the severe acute respiratory syndrome coronavirus 2 (SARS-CoV-2) spike protein with the last 19 aa removed (VSV-SΔ19). The SΔ19 transgene was inserted in VSV between the VSV M and G viral genes. (**b**) The MG1 vector had an enhanced green fluorescent protein reporter transgene (MG1-eGFP). The eGFP transgene was inserted between the G and L viral genes.

More specifically, the VSV vector utilized in this study encodes the gene for a modified version of the SARS-CoV-2 (severe acute respiratory syndrome coronavirus 2) spike protein with the last 19 aa deleted (VSV-SΔ19) (Fig. 1a). This truncated spike protein was used because previous research showed that it enhanced the ability of the spike protein to be included in VSV particles compared to the full-length spike protein [18]. VSV-SΔ19 was originally intended for vaccine-related research but was repurposed for *in vitro* assessment of VSV-mediated oncolysis, where the transgene encoding an antigen should be irrelevant due to the absence of an immune system. Further, the use of transgene-less OVs is becoming progressively rarer in clinical trials. An MG1 engineered to encode an enhanced green fluorescent protein reporter transgene (MG1-eGFP) was also used (Fig. 1b). This transgene was used as a marker to visualize the translational activity of the viral genome in infected cells.

In the case of viral vectors whose bioactivities are hindered at temperatures below that of the body core, adapting them to function well in relatively cold anatomical locations has been shown to be a potential mitigation strategy. For example, a live, attenuated trivalent influenza vaccine was developed for the prevention of influenza A and B [19]. Since it was going to be administered intranasally, both influenza viruses were repeatedly passaged in chicken eggs at gradually decreasing temperatures. This resulted in the generation of cold-restricted variants. This allowed the live-attenuated influenza vaccine to be administered intranasally and its replication was limited to the upper respiratory tract where temperatures do not exceed ~33 °C [19].

The study presented here sought to characterize the effect of temperatures <37 °C on the oncolytic and replicative ability of VSV-SΔ19 and MG1-eGFP viral vectors. Should the replication of the viral vectors be impeded at low anatomical temperatures, we were prepared to attempt the generation of cold-adapted viruses as a potential mitigation strategy. We hypothesized that the viruses would have decreased virus-mediated cell-killing abilities when tested for their oncolytic potential at lower temperatures in cell lines. Consequently, we predicted that cold-adapted versions would be required to outperform their parental counterparts at lower temperatures.

## Methods

### Cell lines and reagents

Vero cells derived from African green monkey kidney epithelial tissue (ATCC CCL-81), HeLa human cervical cancer cells (ATCC CCL-2), murine B16-F10 melanoma cells (ATCC CRL-6475) and A549 human lung adenocarcinoma cells (ATCC CCL-185) were purchased from the American Type Culture Collection (ATCC). Vero and B16-F10 cells were maintained in Dulbecco’s Modified Eagle Medium (HyClone; Cat. no. SH3002201). HeLa cells were maintained in Minimum Essential Medium Eagle (Corning; Cat. no. 10-010-CV). A549 cells were maintained in F-12 Kaighn’s Modification Medium (Cytiva; Cat. no. SH30526.01). All media were supplemented with 10 % heat-inactivated FBS (Corning; Cat. no. 35-087-CV). The cells were cultured in a humidified incubator at 5 % CO_2_ and 37 °C unless otherwise indicated.

### Viruses

Maraba virus with a transgene encoding enhanced green fluorescent protein (MG1-eGFP) was provided by Dr David Stojdl (Children’s Hospital of Eastern Ontario, Ottawa, ON, Canada) [20]. The eGFP transgene was inserted between the G and L viral genes [12]. pVSV Venus VSV-G was a gift from Connie Cepko (Addgene plasmid no. 36399; http://n2t.net/addgene:36399; RRID:Addgene_36399) [21]. The Venus reporter gene was removed using Xhol and Ball restriction enzymes and the pVSV plasmid was re-ligated using infusion cloning. The SARS-CoV-2 SΔ19 transgene was then inserted into the pVSV plasmid between the VSV M and the G viral genes via infusion cloning. The VSV-SΔ19 virus was rescued and propagated as previously described [22]. VSV-eGFP was generously provided by Brian Lichty, McMaster University. VSV-eGFP had the eGFP inserted between the G and L viral genes [23]. The backbone of both VSVs was the Indiana strain.

Viral titres were quantified by either the standard viral plaque assay or utilizing the TCID_50_ assay. For the viral plaque assay, a six-well plate of confluent Vero cells was infected with various dilutions of the virus sample to be titred. The plates were incubated at 37 °C for 1 h and rocked every 10 min. Following this the viral supernatant was aspirated and wells were washed with PBS and a 2 % agarose overlay was added. Plaques were counted 24–36 h later, and viral titre was calculated in plaque-forming units per millilitre (p.f.u. ml^–1^). For the TCID_50_ assay a 96-well plate of confluent Vero cells were infected with serial dilutions of the virus to be titred. Vero cells were infected with each dilution in 12 different wells for greater accuracy when calculating the titre. Each well was checked, via microscopy, for cytopathic effect (CPE) and viral titre was calculated using the Spearman–Kärber method to get the final titre (p.f.u. ml^–1^) [24].

### Cell seeding assays

Cell seeding assays were conducted before any cell viability assays to ensure optimal densities were used. Serial dilutions of cells were seeded into 96-well plate plates in triplicate. After the desired time had passed, resazurin sodium salt (Sigma-Aldrich; Cat. no. R7017) was added at a final concentration of 0.25 mg ml^−1^ and fluorescence was quantified with a plate reader (excitation wavelength: 535/25 nm, emission wavelength: 590/35 nm) 4, 5 and 6 h later. Results were graphed and a one-way ANOVA with Tukey’s multiple comparisons was conducted to determine if there were any significant differences between timepoints following resazurin addition. To identify the optimal seeding density a line of best fit was used to find the *y*-value of the point with maximum fluorescence. Using the appropriate equation based on the line of best fit, 90 % of the calculated *y*-value was used to solve for *x*, which was defined as the optimal seeding density.

Cell seeding densities were optimized for 48 h resazurin dye-based assays (Fig. S1A and B, available in the online ersion of this article). The optimal seeding density was rounded to 8500 cells per well for Vero cells and 5000 cells per well for HeLa and B16-F10 cells, and the optimal time to read the plates following the addition of resazurin was determined to be 5 h. For 72 h resazurin assays (Fig. S1C and D) the optimal seeding density was 1500 cells per well for both Vero and B16-F10 cells and 250 cells per well for HeLa cells, and the plates were read 4 h following the addition of the resazurin dye.

### Cell viability assays

Resazurin assays are relatively high-throughput methods that quantify metabolic activity which correlates with cell viability. They were conducted to investigate the effect of lower temperatures on the oncolytic ability of VSV-SΔ19, VSV-eGFP and MG1-eGFP. The assays were completed at a range of temperatures, including 28, 31, 34 and 37 °C. The effect of decreased temperature was investigated in Vero, B16-F10 and HeLa cells as a strategy to assess whether effects of low temperature treatment might be cell line- and/or species-dependent. Cells were seeded into 96-well plates. The viruses were added at a range of m.o.i. values from 10 to 0.0065 (2.5-fold serial dilution). A schematic of the plate setup is shown in Fig. S2. The plates were incubated at the desired temperatures for 48–72 h. Then resazurin sodium salt was added at a final concentration of 0.25 mg ml^−1^ and fluorescence was quantified with a plate reader 5 and 4 h later for the 48 and 72 h resazurin assays, respectively. Cell viability was calculated as a percentage change relative to untreated control cells, after subtracting background fluorescence, which was based on the average of the wells containing media only. The same controls were incorporated for each cell culture temperature.

### Attempted cold adaptation of VSV-SΔ19 and MG1-eGFP

Two methods were used to attempt cold adaptation of VSV-SΔ19 and MG1-eGFP. The first method was a slow decreasing progression in temperature. For this, the initial infection was completed at 37 °C and the next at 34 °C. From 34 °C, each subsequent infection was completed at half a degree lower temperature for each passage, down to 28 °C for a total of 14 passages. The second method was an immediate drop in temperature, with the first infection occurring at 37 °C, followed by five passages at 31 °C and an additional five at 28 °C. The rationale for not passaging at temperatures between 34 and 37 °C was because oncolytic activity was not significantly abrogated across this range.

**Fig. 2.**
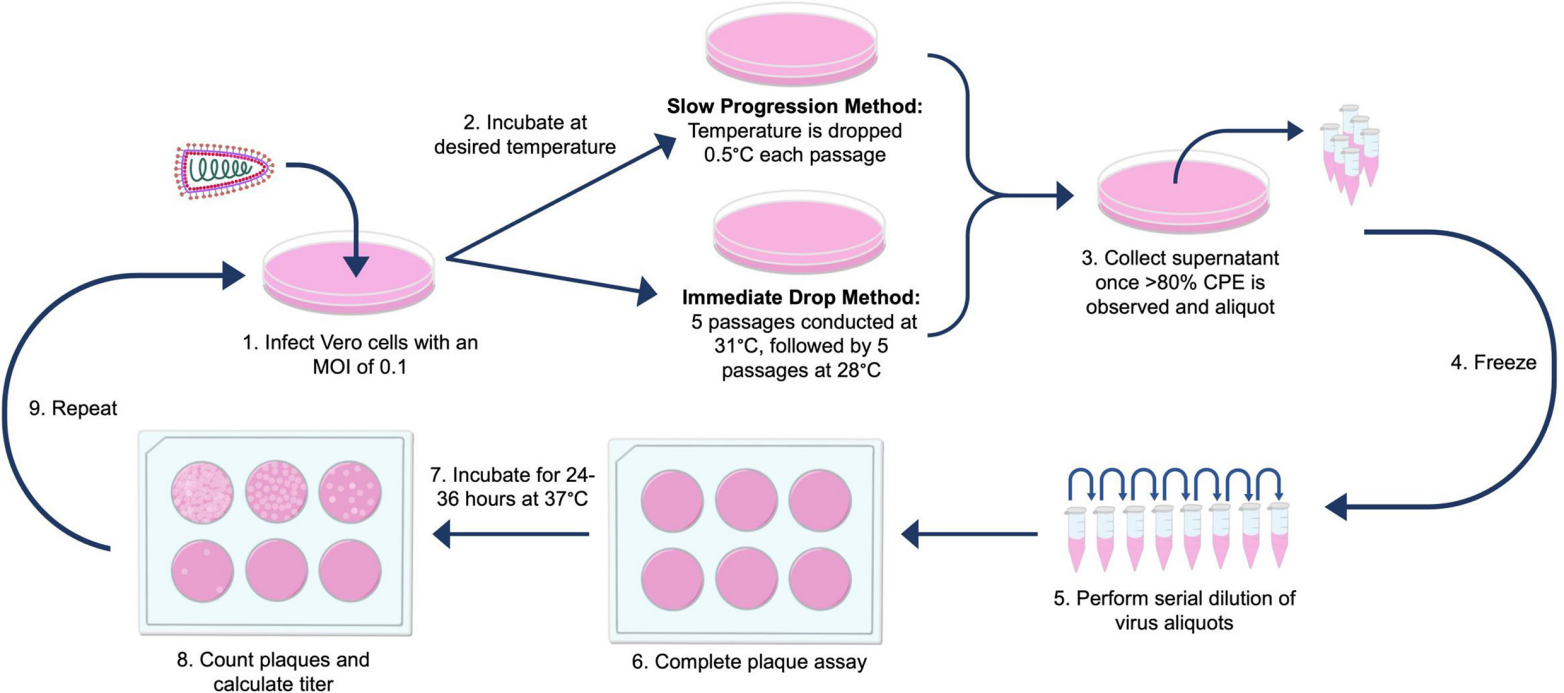
Schematic of the proposed cold-adaptation strategy. Regardless of strategy, the passaging process for cold adaptation was the same. A dish of confluent Vero cells was infected at an m.o.i. of 0.1 and incubated at the desired temperature, depending on the cold-adaptation method used, until >80 % cytopathic effect (CPE) was observed via microscopy. Subsequently, the cells and supernatant were collected and centrifuged. The clarified supernatant was aliquoted and frozen. These aliquots were used to perform plaque assays to determine viral titres (p.f.u. ml^–1^). This process was repeated with each passage.

The infection and titration process for both cold-adaptation strategies were the same (Fig. 2). Parental VSV-SΔ19 and MG1-eGFP were serially passaged in Vero cells at an m.o.i. of 0.1 to allow for multiple rounds of viral replication. Vero cells were used because they are the producer cell line used to propagate these viruses [25]. The infections were incubated at the desired temperature until ≥80 % CPE was observed between 24 and 72 h. Allowing the infections to reach a high level of CPE ensured more time for viral replication and therefore greater chance for an adaptive mutation to occur. Following infection, virus-containing supernatants were collected and centrifuged for 10 min at 700 ***g*** at 4 °C. The supernatants were then stored at −70 °C.

The VSV-SΔ19 and MG1-eGFP that had undergone the ‘immediate drop’ cold-adaptation method and the VSV-SΔ19 that underwent the ‘slow progression’ method were compared with parental viruses to investigate if cold adaptation had occurred. This was done by completing metabolic resazurin assays at 28, 31, 34 and 37 °C in Vero cells. MG1-eGFP was not used at this stage because its titre was too low following the attempt to cold-adapt it via the ‘slow progression’ method.

### Single-step *in vitro* growth curves

To assess the viruses’ replicative ability, single-step *in vitro* growth curves were generated for VSV-SΔ19 and MG1-eGFP. The assay was performed in Vero, B16-F10 and HeLa cells at 28, 31, 34 and 37 °C. Confluent wells were infected with an m.o.i. of 10. This high m.o.i. was used to ensure that each cell was infected with virus at the same time so the effect of decreased temperature on a single cycle of virus replication could be investigated [26]. Plates were incubated at 37 °C for 1 h and were rocked every 10 min. Subsequently, the wells were washed once with PBS and complete medium was added before incubation. The plates were incubated at the desired temperature and virus-containing supernatants were collected at 0, 12, 24, 36, 48 and 60 h time points. Virus-containing supernatants were collected and clarified by centrifugation at 800 ***g*** at 4 °C for 5 min and stored at −70 °C. Once all time points were completed, the samples were titred using the TCID_50_ assay.

### Multi-step virus yield assays

To further investigate the ability of rhabdoviruses to replicate at decreased temperatures, virus yield assays were conducted. Six-well plates of confluent Vero, B16-F10 and A549 cells were infected with VSV-eGFP at an m.o.i. of 0.01. This lower m.o.i. was used to investigate how temperature affected the ability of the viruses to replicate and infect adjacent cells, which is more biologically relevant to clinical applications. Plates were incubated at 37 °C for 1 h and were rocked every 10 min. Then the wells were washed once with PBS and complete medium was added before incubation. The plates were incubated at 28, 31, 34 and 37 °C and virus-containing supernatants were collected at 24 and 48 h time points. Supernatants were centrifuged at 800 ***g*** at 4 °C for 10 min and stored at −70 °C. Once all time points were collected the samples were titred using the standard plaque assay.

### Cell imaging

Vero or B16-F10 cells were left untreated or treated with an m.o.i. of 0.1 of VSV-eGFP. Cells were cultured at 28, 31, 34 or 37 °C. Cells were then treated with a Hoechst 33342 stain (Biotium) and then imaged using a fluorescence microscope at 24 and 48 h post-infection.

### Cell proliferation assay

To identify the effect of decreased temperatures on cellular replication, a cell proliferation assay was completed. For this assay Vero and B16-F10 cells were stained with carboxyfluorescein succinimidyl ester (CFSE) following the protocols outlined previously [27,27]. CFSE forms a covalent bond with long-lived intracellular molecules of the stained cells. When cells divide, the tagged molecules are split equally and each daughter cell becomes half as fluorescent as the parent cell, which can be measured via flow cytometry. After washing out extra CFSE dye, cells were seeded into six-well plates and incubated at 28, 31, 34 and 37 °C. At 0, 24 and 48 h time points, cells were collected, washed with PBS, centrifuged and resuspended in fluorescence-activated cell scanning (FACS) buffer (PBS+0.5 % BSA; Hyclone). All samples were run on a FACSCanto II flow cytometer (BD) and the data were collected using FACS Diva software (version 8.0.1; BD) and analysed using FlowJo v.10.1 (FlowJo).

### Cell death assay

Resazurin dye-based assays provide data that tend to correlate with cell death. Therefore, a flow cytometry assay was used to directly assess the expression of markers of Vero cell death due to treatment with VSV. Vero cells cultured in six-well plates were treated with VSV-eGFP at an m.o.i. of 0.01. Cells were incubated at 28, 31, 34 or 37 °C for 24–48 and 72 h. Following treatment, cells were co-stained with 7-aminoactinomycin D (7-AAD) and annexin V or 7-AAD and anti-caspase-3/7 (all from BioLegend), followed by analysis on a FACS Canto II flow cytometer using FACSDiva 8.0.1 acquisition software. Data were analysed using FlowJo v.10.1.

### Statistical analyses

GraphPad Prism 9 for Mac OS (GraphPad software) was used for all graphing and statistical analysis. Data from metabolic resazurin assays, single-step *in vitro* growth curves and cell proliferation assays, which involved two variables, were assessed by two-way ANOVA with Dunnett’s multiple comparisons test. An ordinary one-way ANOVA with Dunnett’s multiple comparisons was used to analyse the cell seeding assay data and the multi-step virus yield assay data. All reported *P-*values were two-sided and were considered significant at *P*≤0.05. Graphs show means and standard errors.

## Results

### The oncolytic ability of VSV-SΔ19 and MG1-eGFP was hindered at lower temperatures

The primary objective of this study was to use *in vitro* cell culture-based modelling to determine whether relatively low temperatures could be of clinical relevance to the use of viral vectors. Importantly, viral vectors such as OVs have limited therapeutic windows *in vivo,* due to the induction of innate and adaptive immune responses. In particular, innate immune responses can eliminate these viruses from the body within 48 h [28,32]. As such, initial *in vitro* assessments of the efficacy of viruses were limited to a 48 h time window.

**Fig. 3.**
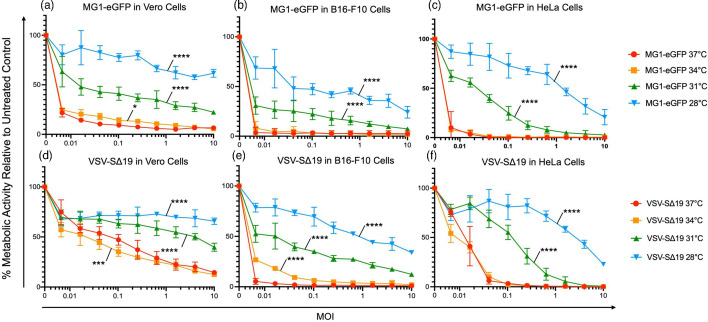
The effect of decreased temperature on the oncolytic ability of Maraba virus (MG1-eGFP) and vesicular stomatitis virus (VSV-SΔ19). Graphs showing the results of 48 h metabolic resazurin assays for MG1-eGFP in (**a**) Vero, (**b**) B16-F10 and (**c**) HeLa cells, and VSV-SΔ19 in (**d**) Vero, (**e**) B16-F10 and (**f**) HeLa cells at 28, 31, 34 and 37 °C. Statistical analysis was performed using a two-way ANOVA with Dunnett’s multiple comparisons. Means and standard errors are shown (*n*=3; ****P<0.0001, ***P<0.001, *P<0.05; all data sets are compared to the 37 °C curves).

To assess the effect of low temperatures on the oncolytic potential of MG1-eGFP and VSV-SΔ19, a resazurin dye-based metabolic assay was conducted at 28, 31, 34 and 37 °C in Vero, B16-F10 and HeLa cells. In all three cell lines, the oncolytic activity of MG1-eGFP and VSV-SΔ19 was significantly abrogated at 28 and 31 °C (*P*<0.0001) (Fig. 3). The effect of 34 °C on the oncolytic ability of MG1-eGFP and VSV-SΔ19 varied in a cell line-dependent manner, with MG1-eGFP and VSV-SΔ19 hindered in Vero cells (*P*<0.05) (Fig. 3a) and B16-F10 cells, respectively (*P*<0.0001) (Fig. 3e). Interestingly, in Vero cells at 34 °C, the oncolytic ability of VSV-SΔ19 was enhanced (*P*<0.001) (Fig. 3d). These results suggest that the oncolytic capabilities of MG1-eGFP and VSV-SΔ19 were consistently hindered at temperatures ≤31 °C.

To confirm these results, images were taken of VSV-SΔ19 and MG1-eGFP-infected monolayers of Vero, B16-F10 and HeLa cells at 28, 31, 34 and 37 °C. Cells were infected at an m.o.i. of 0.1 and photos were taken with a brightfield microscope at 48 h post-treatment (Fig. S3). The photos showed that both viruses caused visibly less CPE at lower temperatures. Specifically, the higher temperatures had a greater number of rounded cells and more had lifted off the surface of the wells compared to the plates incubated at lower temperatures.

These findings suggested that the oncolytic ability of VSV-SΔ19 and MG1-eGFP was hindered at lower temperatures, which could have negative implications when using these viruses as therapeutics at anatomical sites <37 °C.

### Attempted cold adaptation of VSV-SΔ19 and MG1-eGFP

Since the virus-mediated cell killing ability of VSV-SΔ19 and MG1-eGFP proved to be impeded at decreased temperatures, cold adaptation was attempted as a mitigation strategy. This approach was undertaken because unpublished data from our research group showed that rhabdoviral vectors could be heat-adapted to increase their oncolytic ability at higher temperatures. So, the same approach was used here but for lower temperatures.

Cold adaptation was attempted by serially passaging VSV-SΔ19 and MG1-eGFP at lower temperatures using two strategies. The first method, termed ‘slow progression’, involved a gradual decrease in the temperature across 14 passages. The second method was referred to as the ‘immediate drop method’ and it utilized two rapid decreases in temperature, with multiple passages at each temperature. Virus-containing supernatants from each infection were titred following each passage (Fig. S4).

For VSV-SΔ19, the viral titres appeared stable for both the slow progression and immediate drop methods. There was variability in titres for the VSV-SΔ19 that underwent the slow progression method, but this could be attributed to the inherent variability of the standard plaque assay, the process of infecting cells and collecting supernatants.

MG1-eGFP that underwent both methods followed a declining trend, with virus titres decreasing by several log towards the end of the treatment. By the 30 °C passage of the slow progression method and the fourth 28 °C passage of the immediate drop method, the titre of MG1-eGFP had become too low to complete the next passage while maintaining an m.o.i. of 0.1. This could have been due to the production of defective interfering particles (DIPs).

DIPs have defective viral genomes that are lacking a part of the genome needed for completion of the viral life cycle [33]. DIPs require infectious viral particles to replicate and if both infect a cell DIPs will block production of the standard infectious particles. They are produced through normal replication of negative stranded RNA viruses so it was expected in a protocol, such as the one we used here, where viruses were being repeatedly passaged that DIPs would be formed [34]. Furthermore, since they interfere with wild-type virus replication they may also alter plaque assay results. To mitigate this, plaque purification was done for an infectious MG1-eGFP virus clone from the 30 °C passage of the slow progression method and the fourth 28 °C passage of the immediate drop method. This resulted in a several log increase in viral titre for the MG1-eGFP that underwent the immediate drop method. This recovery was not as substantial for the MG1-eGFP that underwent the slow progression method. Plaque purification recovered the viral titre sufficiently to complete the passages until 28 °C. However, by that passage the viral titre was again too low to continue with any subsequent experiments.

To determine if cold-adapted viruses were generated, the virus-mediated cell killing ability of VSV-SΔ19 and MG1-eGFP that had been passaged under a cold selective pressure were compared to the parental viruses via metabolic resazurin assays at 28, 31, 34 and 37 °C in Vero, B16-F10 and HeLa cells. The assays were conducted for VSV-SΔ19 that underwent both methods, but only for MG1-eGFP that underwent the immediate drop method, since titres became too low with the second method (Fig. S4).

**Fig. 4.**
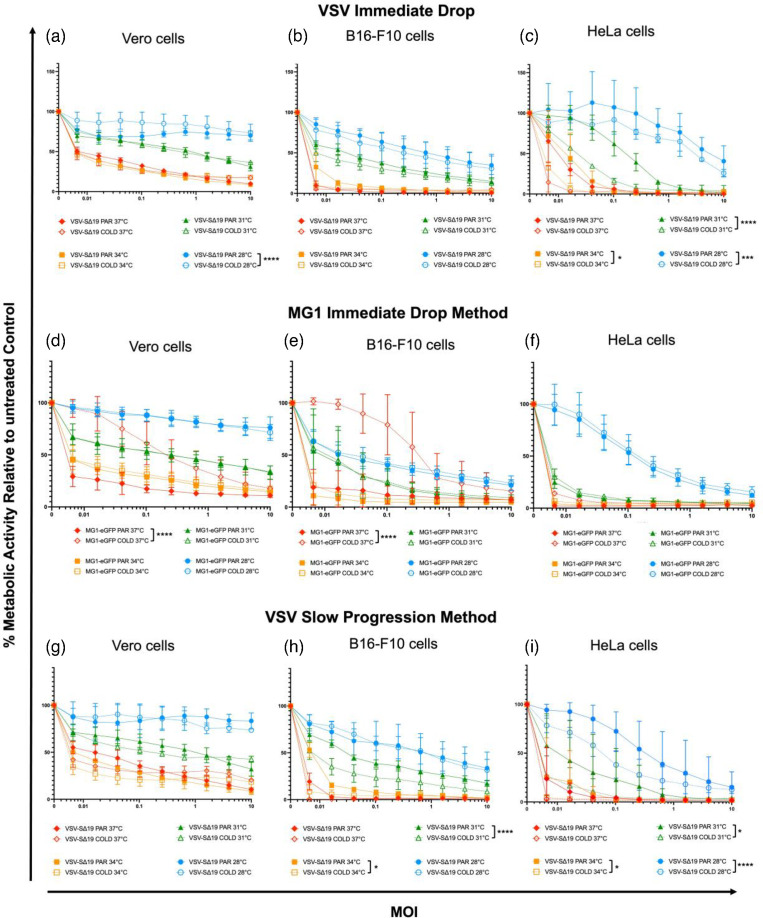
Comparing the oncolytic activities of Maraba virus (MG1-eGFP) and vesicular stomatitis virus (VSV-SΔ19) for which cold adaptation was attempted. Graphs show the results of 48 h metabolic resazurin assays at 28, 31, 34 and 37 °C for the parental viruses and VSV-SΔ19 that underwent the ‘immediate drop’ cold adaptation method in (**a**) Vero cells, (**b**) B16-F10 cells and (**c**) HeLa cells, MG1-eGFP that underwent the ‘immediate drop’ cold adaptation method in (**d**) Vero cells, (**e**) B16-F10 cells and (**f**) HeLa cells, and VSV-SΔ19 that underwent the ‘slow progression’ cold adaptation method in (**g**) Vero cells, (**h**) B16-F10 cells and (**i**) HeLa cells (*n*=3, ****P<0.0001, ***P<0.001, *P<0.05; all data sets are compared to the 37 °C curves).

For the VSV-SΔ19 that underwent the ‘immediate drop’ method, in Vero cells, the parental virus had greater cell-killing ability at 28 °C (*P*<0.0001). This means the virus may have been hindered in its ability to mediate CPE at lower temperatures in the Vero cell line following cold adaptation (Fig. 4a). There was no benefit nor hindrance to oncolytic activity in B16-F10 cells (Fig. 4b). The vector only had significantly better cell-killing ability at 34 °C (*P*<0.05), 31 °C (*P*<0.0001) and 28 °C (*P*<0.001) in HeLa cells (Fig. 4c).

The MG1-eGFP that underwent the ‘immediate drop’ selection method had significantly worse cell-killing ability at 37 °C in Vero and B16-F10 cells (*P*<0.0001) (Fig. 4d, e, respectively). Again, this suggested that the efficacy of MG1-eGFP had been impeded and possibly cold-restricted. There was no benefit nor hindrance to oncolytic activity in HeLa cells (Fig. 4f).

Lastly, for the VSV-SΔ19 that underwent the ‘slow progression’ method there was no benefit in producer Vero cells (Fig. 4g). There was significantly greater cell-killing ability at 34 °C (*P*<0.05) and 31 °C (*P*<0.0001) in B16-F10 cells (Fig. 4h) and at 34 °C (*P*<0.05), 31 °C (*P*<0.001) and 28 °C (*P*<0.0001) in HeLa cells (Fig. 4i).

The intention of cold adaptation was to produce cold-adapted mutants of VSV-SΔ19 and MG1-eGFP that functioned better at lower temperatures compared to their parental counterparts and maintain functionality at 37 °C across multiple cell lines. From these results it is evident that the viruses did not become sufficiently cold-adapted.

### Viral replication was not hindered at lower temperatures

**Fig. 5.**
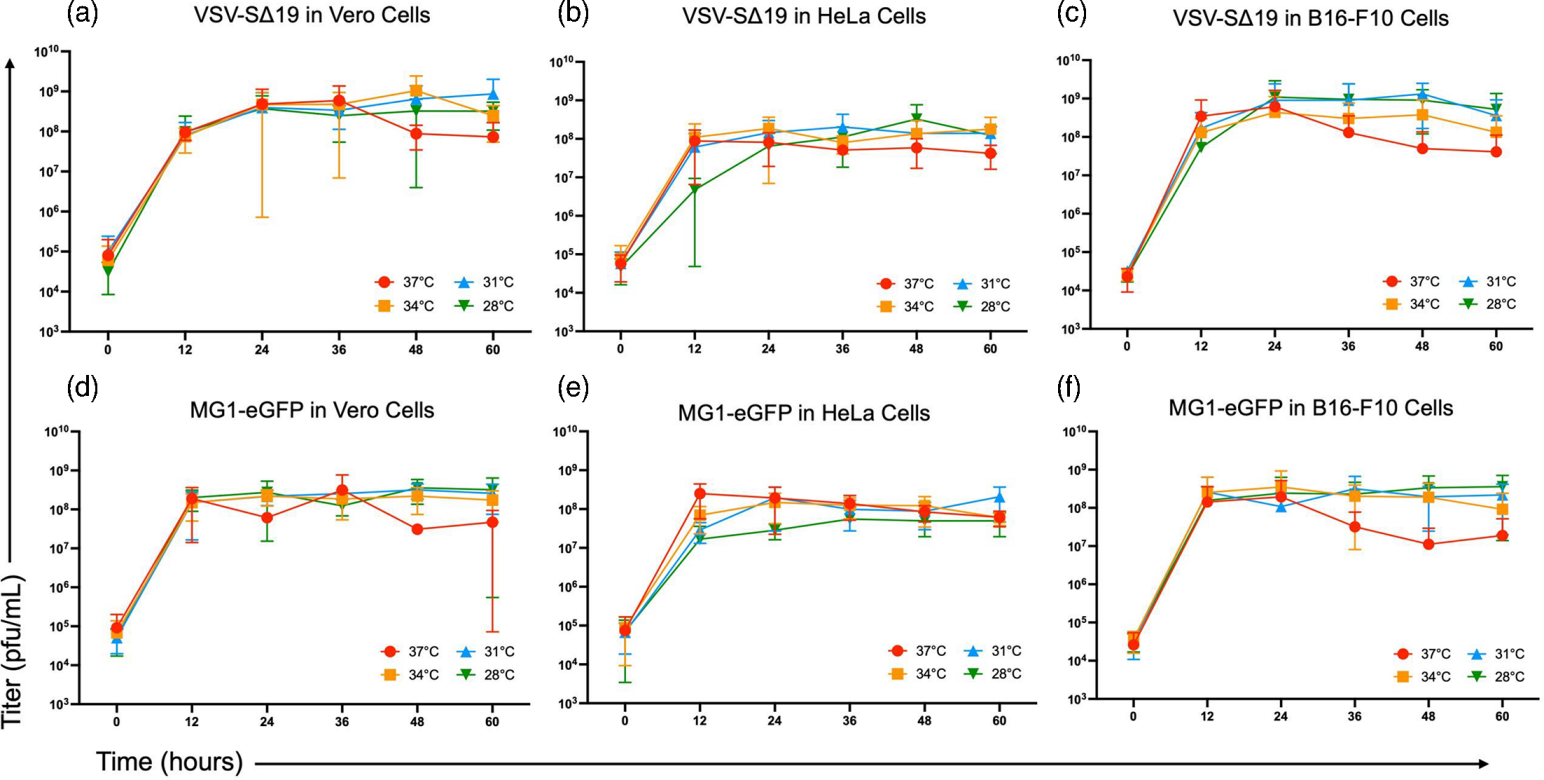
The effect of temperatures <37 °C on the replicative ability of Maraba virus (MG1-eGFP) and vesicular stomatitis virus (VSV-SΔ19). Graphs showing the results of 60 h single-step *in vitro* growth curves for MG1-eGFP in (**a**) Vero, (**b**) B16-F10 and (**c**) HeLa cells, and VSV-SΔ19 in (**d**) Vero, (**e**) B16-F10 and (**f**) HeLa cells at 28, 30, 34 and 37 °C. Viruses were titrated using the TCID_50_ assay and results were expressed in plaque-forming units per millilitre (p.f.u. ml^–1^). Statistical analysis was performed using a two-way ANOVA with Dunnett’s multiple comparisons. Means and standard errors are shown. There were no significant differences between any of the curves (*n*=3 for Vero and B16-F10 cells, *n*=2 for HeLa cells).

Since the proposed cold-adaptation strategies did not produce sufficiently cold-adapted viral mutants, the replicative ability of the viruses was investigated to potentially understand why they could not be cold-adapted. First, single-step *in vitro* growth curves were conducted to identify if temperatures <37 °C affected the ability for viruses to infect and/or replicate in cells. Vero, B16-F10 and HeLa cells were treated with VSV-SΔ19 and MG1-eGFP at an m.o.i. of 10 and incubated at 37, 34, 31 and 28 °C. Virus-containing supernatants were collected at 0, 12, 24, 36, 48 and 60 h and titres were determined using the TCID_50_ assay. There was no significant difference in viral titres between the different temperatures for either virus in any of the cell lines (Fig. 5).

Since the single-step *in vitro* growth curves utilized a high m.o.i., they only showed the effect of lower temperatures on a single virus replication cycle, excluding viral entry. To investigate if the lower temperatures affected the ability of the viruses to infect an initial cell, replicate and then infect other cells, multi-step virus yield assays were conducted. These assays were completed in Vero, B16-F10 and A549 cells at 37, 34, 31 and 28 °C.

Only VSV-eGFP was used in this and all subsequent assays instead of VSV-SΔ19 and MG1-eGFP. This was because VSV-SΔ19, which was being developed as an intranasal vaccine for COVID-19, did not demonstrate any substantial cold adaptation. Further, the MG1-eGFP had not performed as well as VSV in the cold-adaptation process. So, the focus turned to mechanistic studies using VSV-eGFP that had undergone parallel failed attempts at cold adaptation. This allowed for imaging following viral infections.

**Fig. 6.**
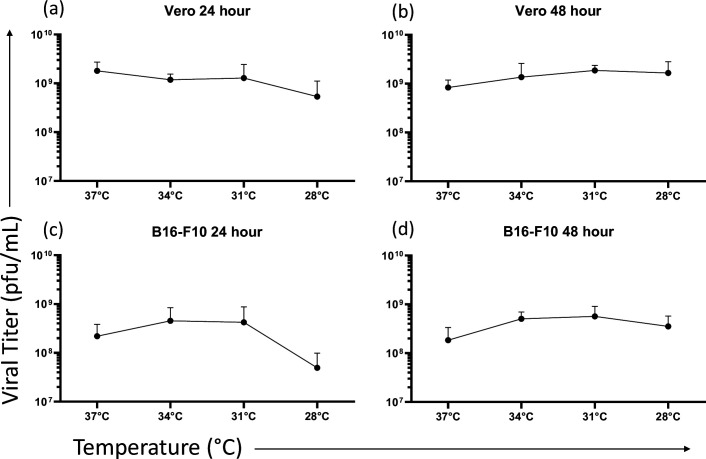
The effect of temperatures <37 °C on the replicative ability of vesicular stomatitis virus (VSV-eGFP). Graphs showing the results of virus yield assays conducted in Vero cells at (**a**) 24 and (**b**) 48 h and B16-F10 cells at (**c**) 24 and (**d**) 48 h. Cells were infected at an m.o.i. of 0.01 and incubated at 28, 31, 34 and 37 °C. Viral supernatants were collected at 24 and 48 h time points and titrated using the standard plaque assay and are in plaque forming units per millilitre (p.f.u. ml^–1^). Statistical analysis was performed using a one-way ANOVA with Tukey’s multiple comparisons. For both time points, none of the viral titres at temperatures <37 °C differed from the respective titre at 37 °C. Means and standard errors are shown (*n*=3 per treatment).

Following treatment of cells to generate multi-step growth curves, virus-containing supernatants were collected at 24 and 48 h and titred using the standard plaque assay. Results were graphed and were similar to the results of the single-step *in vitro* growth assays (Figs 6 and S5). There were no significant differences in the final viral titres at each of the temperatures, in any of the cell lines, further suggesting that lower temperatures do not affect overall virus production capabilities.

**Fig. 7.**
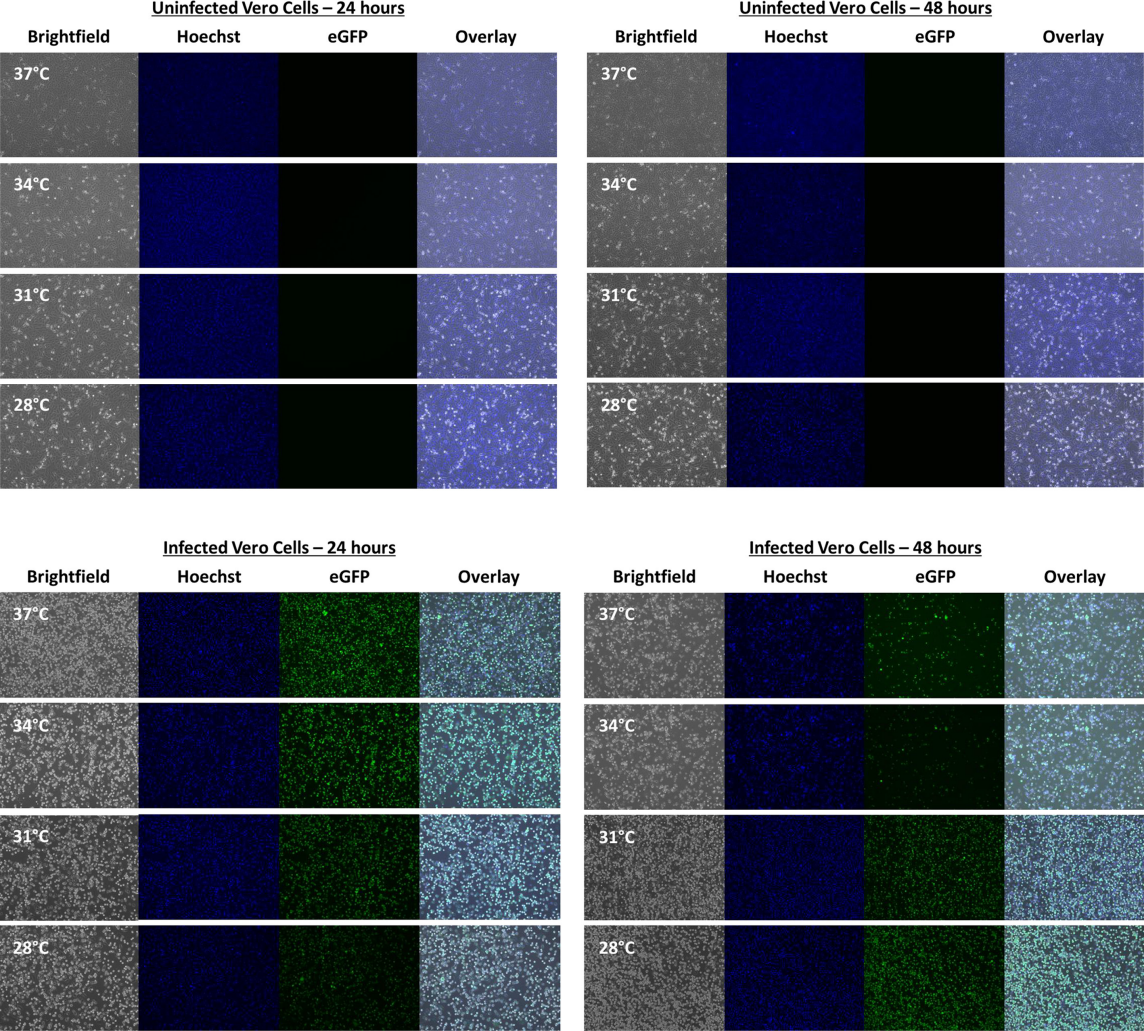
The effect of decreased temperature on the virus-mediated cell killing ability of vesicular stomatitis virus (VSV-eGFP). Photos taken with a fluorescence microscope. Representative brightfield images (photos on the left), and images of Hoechst staining (middle left) and green fluorescence (middle right) are shown alongside an overlay of all three (photos on the right). Photos are of Vero cell cultures in six-well plates that were treated with VSV-eGFP at an m.o.i. of 0.01 at 28, 31, 34 and 37 °C for 24 and 48 h.

Photos were also taken following infection of cells for the multi-step virus-yield assay to observe changes in cell morphology and the relative amount of eGFP (Fig. 7). Photos taken by brightfield microscopy showed a greater number of rounded and lifted cells, indicative of greater CPE, at 37 °C compared to lower temperatures for Vero cells. The amount of CPE at all temperatures was greater at 48 h compared to 24 h, but at 48 h CPE was still greatest at 37 °C.

A similar imaging experiment was repeated for Vero cells but included a head-to-head comparison with B16-F10 cells (Fig. S6). Again, photos taken by brightfield microscopy showed a greater number of rounded and lifted cells, indicative of greater CPE, at 37 °C compared to lower temperatures for both Vero and B16-F10 cells. For Vero cells, the relative amount of eGFP at 24 h, as assessed by microscopy, appeared to be similar at 37, 34 and 31 °C with visibly less eGFP only at 28 °C. In B16-F10 cells, at 24 h, there was slightly less eGFP with each drop in temperature. Notably, at 48 h at 28 °C, both cell lines seemed to have comparable amounts of CPE to the infections at 37 °C at 24 h. This suggests that the kinetics of virus transgene expression are affected by temperature, but the overall and absolute transgene expression levels are equal given enough time.

### Rate of cell proliferation was hindered at lower temperatures

Since lower temperatures seemed to affect the oncolytic potential and the transgene kinetics of the viruses tested but not their absolute virus yields or transgene expression, the effect of lower temperatures on the cells was investigated as a potential explanation for this discrepancy. To accomplish this, flow cytometry was used to analyse the rate of cell proliferation of Vero and B16-F10 cells at 37, 34, 31 and 28 °C using the fluorescent cell proliferation dye CFSE. Cells were stained and analysed at 24, 48 and 72 h.

**Fig. 8.**
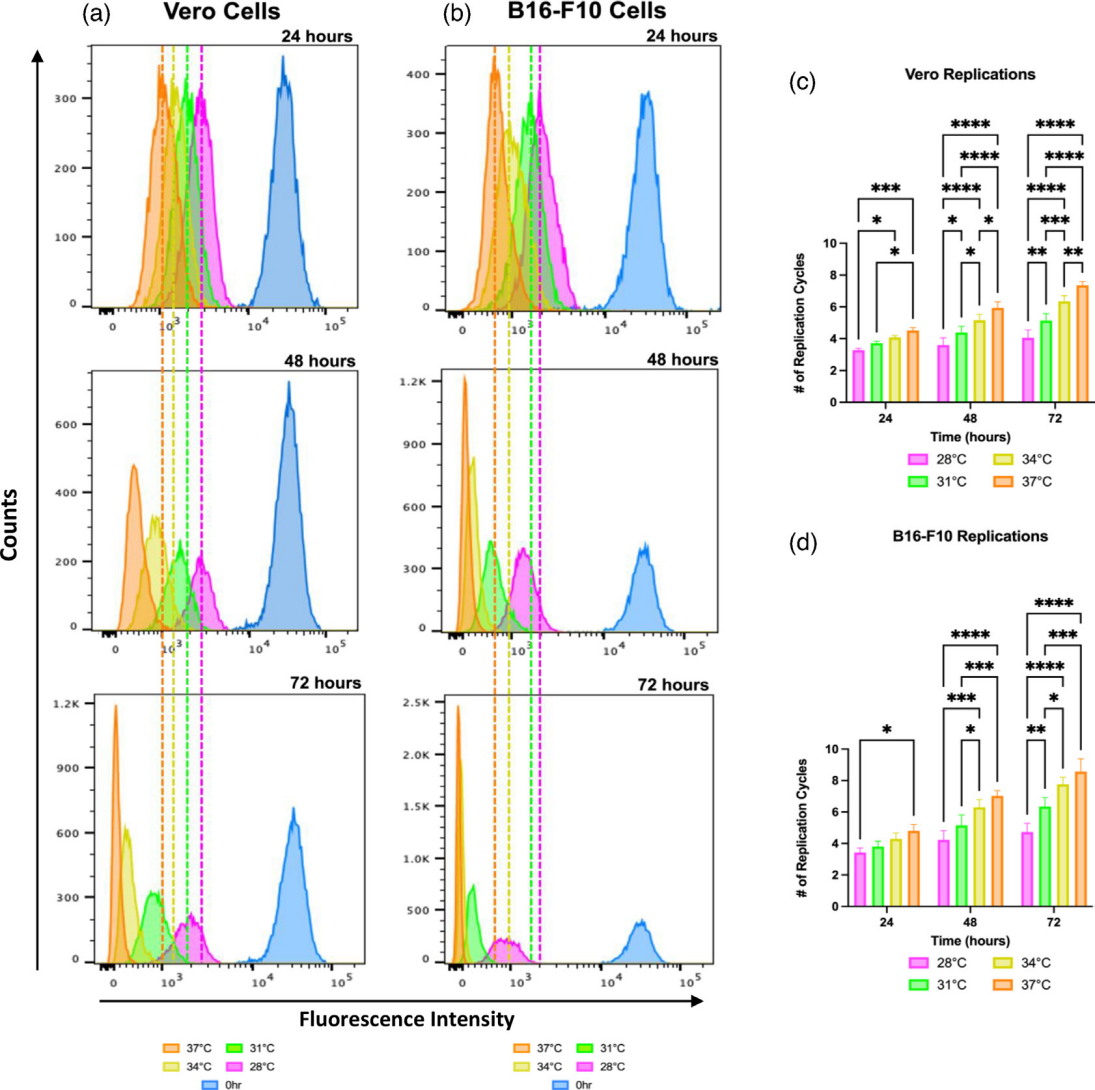
The effect of temperatures <37 °C on cell proliferation. Vero and B16-F10 cells were stained with the fluorescent proliferation dye CFSE and then incubated at 28, 31, 34 and 37 °C. Fluorescence was analysed via flow cytometry at 0, 24, 48 and 72 h. Representative histograms show the fluorescence of CFSE (*x*-axes) of (**a**) Vero and (**b**) B16-F10 cells at 24, 48 and 72 h. Geometric means of CFSE fluorescence intensity were used to calculate the number of (**c**) Vero and (**d**) B16-F10 replication cycles relative to the 0 h time point for each temperature at 24, 48 and 72 h. Statistical analysis was performed using a two-way ANOVA with Tukey’s multiple comparisons. Means and standard errors are shown (*n*=3 per treatment; ****P<0.0001, ***P<0.001, **P<0.01, *P<0.05).

Histograms depicting the fluorescence of the Vero and B16-F10 cells grown at 37, 34, 31 and 28 °C and collected at 24, 48 and 72 h are shown in Fig. 8a, b. The proliferation of both Vero and B16-F10 cells correlated with a reduction in temperature. For Vero cells, it took 48 h at 34 °C and 72 h at 31 °C to proliferate as much as the same cells at 37 °C for 24 h. For B16-F10 cells similar trends were seen. However, B16-F10 cells went through more cell cycles over the same time span as compared to Vero cells. This was expected and acted as an internal control since B16-F10 cells are known to replicate faster than Vero cells [35,36].

The geometric mean fluorescence intensity for the cells incubated at each temperature and time point were used to calculate the number of cell divisions completed compared to 0 h controls (Fig. 8c, d). For both Vero and B16-F10 cells, at all temperatures and all time points there was a significant difference between the number of cell cycles at 37 and 28 °C. This indicated that lower temperatures slowed the rate of cell proliferation, which could have impacted the oncolytic activity of viruses in the resazurin assays.

### Kinetics of rhabdovirus cell killing is hindered by reduced temperatures

**Fig. 9.**
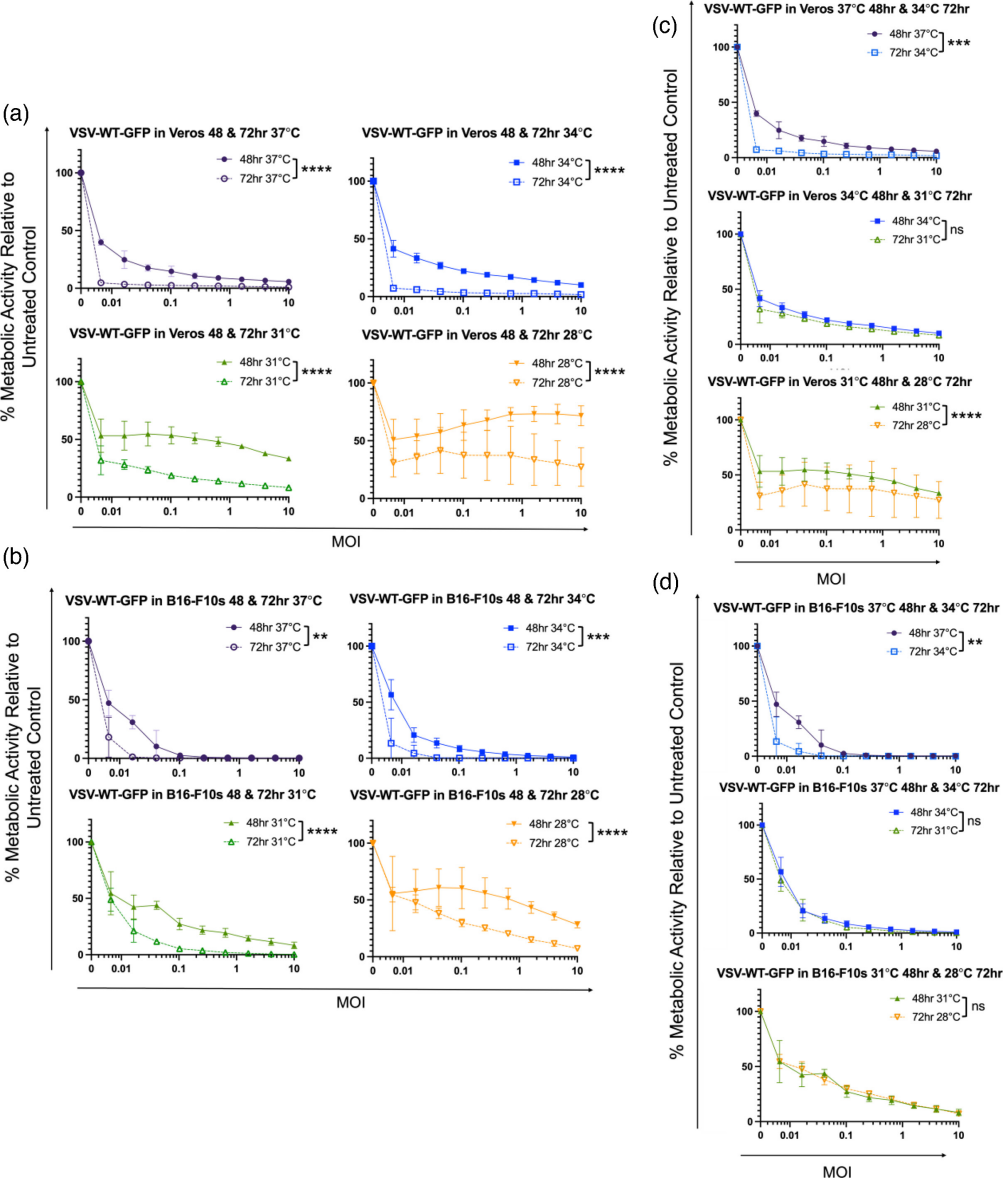
The effect of temperatures <37 °C on vesicular stomatitis virus (VSV-eGFP)-mediated cell killing at 48 and 72 h. Graphs showing the results of 48 and 72 h metabolic resazurin assays for VSV-eGFP in (**a and b**) Vero cells and (**c and d**) B16-F10 cells at 28, 31, 34 and 37 °C. Statistical analysis was performed using a two-way ANOVA with Tukey’s multiple comparisons. Means and standard errors are shown.

**Fig. 10.**
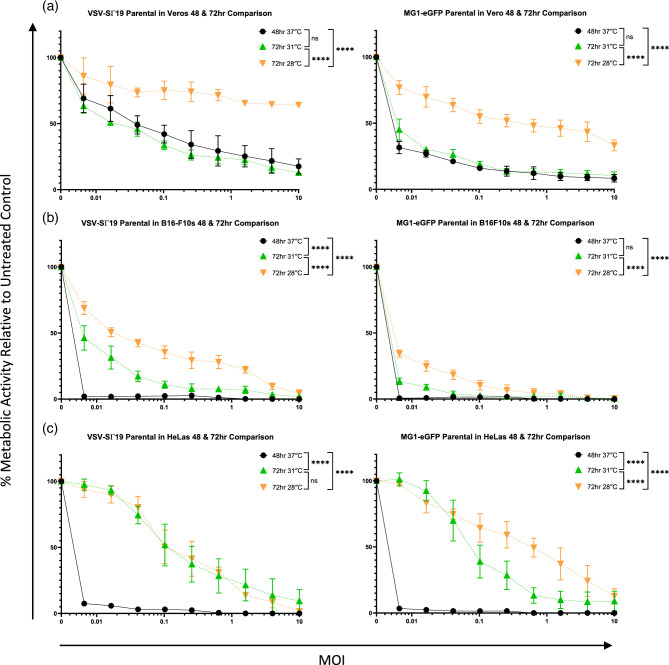
Oncolytic activity of parental VSV-S∆19 and MG1-eGFP in the 48 and 72 h resazurin assays. Graphs showing the results of the 48 and 72 h metabolic resazurin assays for VSV-S∆19 and MG1-eGFP in (**a**) Vero, (**b**) B16-F10 and (**c**) HeLa cells. Each graph shows the comparison of the 48 h 37 °C, 72 h 31 °C and 72 h 28 °C conditions. Statistical analysis was performed using a two-way ANOVA with Tukey’s multiple comparisons. Means and standard errors are shown.

Results of the cell proliferation assays demonstrated that lower temperatures slowed cell cycling. However, they also showed that with more time the cells incubated at lower temperatures could go through similar numbers of cell cycles as those at 37 °C. To investigate the relationship between cell proliferation and virus-mediated cell killing, resazurin assays were conducted using VSV-eGFP in Vero and B16-F10 cells (Fig. 9) and VSV-S∆19 and MG1-eGFP in Vero, B16-F10 and HeLa cells (Fig. 10) at 37, 34, 31 and 28 °C. Assays were run for both 48 and 72 h to assess whether an additional 24 h would potentiate oncolytic activity.

For VSV-eGFP in Vero and B16-F10 cells, at each temperature, there was significantly less metabolic activity at 72 h compared to 48 h (Fig. 9a, b) meaning that with more time the virus continued to infect and kill cells at any temperature.

To assess whether oncolysis at a lower temperature for a longer period of time was comparable to a higher temperature for a shorter period of time, graphs were made to compare two temperatures, the higher at the 48 h time point and the lower at the 72 h time point. For instance, in Vero cells, those incubated at 37 °C for 48 h and at 34 °C for 72 h were shown to be significantly different (*P*<0.0001) with the cells incubated at 34 °C for 72 h having greater virus-mediated cell killing ability than those at 37 °C for 48 h (Fig. 9c, top graph). The same trend was seen with the Vero cells at 31 °C for 48 h and 28 °C for 72 h (*P*<0.0001) (Fig. 9c, bottom graph).

The B16-F10 cells incubated at 37 °C for 48 h and 34 °C for 72 h were also shown to be significantly different, with greater virus-mediated cell killing for the cells incubated at 34 °C for 72 h (*P*<0.01) (Fig. 9d, top graph).

There was no significant difference between the metabolic activity of the Vero cells incubated at 34 °C for 48 h and those incubated at 31 °C for 72 h (Fig. 9b, middle graph). There was also no significant difference between the B16-F10 cells incubated at 34 °C for 48 h and 31 °C for 72 h (Fig. 9d, middle graph) and those incubated at 31 °C for 48 h and 28 °C for 72 h (Fig. 9d, bottom graph).

Expanding this investigation to VSV-S∆19 and MG1-eGFP, it was discovered that parental VSV-S∆19 had equivalent oncolytic activity in a 72 h incubation at 31 °C as it did in a 48 h incubation at 37 °C in Vero cells (Fig. 10a). Similarly, we found that the oncolytic activity of MG1-eGFP in a 72 h incubation at 31 °C was equivalent to its oncolytic activity in a 48 h incubation at 37 °C in both Vero and B16-F10 cells (Fig. 10a, b). Despite an increase in incubation time, the oncolytic activity of both viruses at 72 h at 28 °C in Vero and B16-F10 cells was significantly less than the other conditions (*P*<0.0001). Furthermore, the oncolytic activity of both viruses at 72 h at both 31 and 28 °C in HeLa cells were significantly less than at 37 °C (*P*<0.0001).

Altogether, this showed that in certain cell lines, an equal amount of virus-mediated cell killing, or greater, could be achieved if given more time *in vitro*, where there were no limitations on the therapeutic window.

### VSV-induced apoptosis of cells was delayed when culture temperatures were reduced

The resazurin dye-based assay used to assess viral oncolysis (see Figs3  4) tends to correlate with cell viability but cannot differentiate between cytostatic versus cytopathic effects. Flow cytometric analysis of cell proliferation shown in Fig. 8 demonstrated a reduction in the rate at which cells divided that correlated with a decrease in culture temperatures. To directly assess cytopathic effects of VSV at reduced cell culture temperatures, flow cytometry was used to analyse the expression of markers of cell death due to treatment with VSV. Vero cells were treated with VSV-eGFP at an m.o.i. of 0.01 and then incubated at 28, 31, 34 or 37 °C for 24–48 and 72 h. Following treatment, cells were co-stained with 7-AAD and annexin V or 7-AAD and anti-caspase-3/7. The results shown in Fig. S7 showed that apoptosis of cells was delayed as the temperature decreased. Over time, however, the proportion of apoptotic cells in cultures at lower temperatures caught up to the percentage of cell death observed at earlier time points in cells cultured at higher temperatures.

## Discussion

There is a paucity of research investigating the relationship between viral vector performance and temperatures <37 °C. This could be critically important when utilizing viral vectors as OVs to treat superficial tumours. This study aimed to examine the effect of decreasing temperature on rhabdoviral vector performance, and attempts were made to cold-adapt the viruses as a mitigation strategy. Metabolic resazurin assay results demonstrated that decreasing temperatures hindered rhabdovirus-mediated cell killing. However, two cold-adaptation strategies proved unsuccessful in creating sufficiently cold-adapted mutants for two different rhabdoviruses expressing two different transgenes, VSV-SΔ19 and MG1-eGFP. The reduction in oncolytic activity at lower temperatures suggested that this was due to a concomitant reduction in virus replication cycles. However, with further investigation we demonstrated that decreased temperatures did not affect the replication of either virus but did significantly reduce cell proliferation. VSV-SΔ19 was originally intended for use as an intranasal vaccine. However, since substantial cold adaptation was not achieved, *in vivo* studies were not pursued. Instead, the SΔ19 transgene was replaced with eGFP to facilitate *in vitro* mechanistic studies, with these data beginning at Fig. 6. Additional metabolic resazurin assays showed that with increased time the viruses could achieve the same degree of virus-mediated cell killing at decreased temperatures as those at 37 °C. As such, the reduction in oncolysis was not due to an inherent problem with the rhabdoviruses (i.e. their replication potential was not cold-restricted). Instead, the reduced oncolysis was apparently due to cells becoming more resistant to death at lower temperatures, despite viral titres remaining high.

In the literature, it has been shown that cells cultured at lower temperatures (30 °C) arrest in the G1 phase of the cell cycle and there is a delay in the onset of apoptosis [37]. Also, many RNA viruses induce cell cycle arrest in G1, S or G2 phase to favour viral replication [38]. Therefore, we hypothesize that the cells incubated at the lower temperatures were stuck in G1 phase, which is favourable for viral replication, which could explain why the decreased temperatures did not affect viral replication. Additionally, since temperature reduction delayed apoptosis (see Fig. S6), the cells did not die as quickly at lower temperatures compared to those at 37 °C. This could provide a rationale as to why with more time the viruses mediated equivalent cell killing regardless of temperature.

This poses an issue when using these viruses in a clinical setting. Unlike our *in vitro* model, viral vectors have a limited therapeutic window in clinical settings before they are cleared by the immune system [28,29]. Host antiviral immunity is rapidly activated following viral vector administration which can restrict replication and dissemination of the virus, thereby diminishing the therapeutic effect [39]. For example, antiviral antibodies, complement activation and antiviral cytokines can hinder the efficacy of viral vectors [29,40, 41]. Since we demonstrated that rhabdoviruses require more time to achieve efficacy at lower temperatures that is equivalent to that at 37 °C, this would also allow more time for the host immune system to clear the virus before it reaches its full therapeutic potential. It would also result in the release of fewer tumour-associated antigens over time for tumours at relatively low anatomical temperatures.

Besides cold adaptation, there are other avenues that could be investigated to overcome these hurdles. Methods such as encapsulating the viruses, using carrier cells, or using polymeric materials to mask viral surface proteins can be used to limit detection of the virus by the host immune system and, therefore, buy more time for oncolysis [42,45]. An additional strategy could be the use of hyperthermic medicine, which has been investigated in models of ovarian cancers [46]. A combination of hyperthermic treatment and viral vectors could circumvent the limiting factor of relatively low anatomical temperatures by bringing the temperature closer to the baseline of 37 °C.

A limitation of this study was that only two methods were attempted for cold adaptation. Other approaches could be attempted to produce cold-adapted mutants of the viruses. We previously had success in heat adapting rhabdoviruses to efficiently replicate at temperatures of up to 40 °C. Alternatively, it may be possible to identify rhabdoviruses that naturally replicate at temperatures below 28 °C, such as those from fish. These rhabdoviruses that naturally replicate at very low temperatures could potentially be heat-adapted to efficiently replicate at 28 °C. Cold adaptation can also be approached with more tightly regulated control measures. This could include a 37 °C control running in parallel with each passage completed at a lower temperature for the purpose of identifying relevant mutations in the viral genome should a robustly cold-adapted variant be generated. Or incubation times could be limited to 24 h for each passage to avoid virus degradation or the production of DIPs. Approaches such as larger culture size could also be employed to increase the probability of an adaptive mutant arising and propagating. Additionally, the relationship between temperature, viral replication and cell replication is complex. Further studies could be attempted to more accurately identify the reasons why lower temperatures affect virus-mediated cell killing but not viral replication. This could include identifying the dynamics of cell cycling at the different temperatures as well as during viral infection.

In conclusion, we have identified a novel impediment to the clinical application of rhabdovirus-based vectors. Specifically, we demonstrated that temperatures <37 °C could limit the efficacy of rhabdoviruses by prolonging the time required for them to mediate the same degree of virus-mediated cell killing as at 37°C. This would be expected to limit the efficacy of rhabdoviral vectors in the clinic where the luxury of extending the time that cells are exposed to viruses is lacking due to robust and rapid host immune responses that clear these vectors, thereby limiting the therapeutic window.

## supplementary material

10.1099/jgv.0.002010 Uncited Fig. S1.

## Abbreviations

7-AAD: 7-aminoactinomycin D
CFSE: carboxyfluorescein succinimidyl ester
CPE: cytopathic effect
DIP: defective interfering particle
eGFP: enhanced green fluorescent protein
FACS: fluorescence-activated cell scanning
MG1: Maraba virus
OV: oncolytic virus
SARS-CoV-2: severe acute respiratory syndrome coronavirus 2
VSV: vesicular stomatitis virus
