# Supplementary material for: Investigating the effect of reduced temperatures on the efficacy of rhabdovirus-based viral vector platforms
Source: J Gen Virol. 2024 Aug 22;105(8):002010. doi: 10.1099/jgv.0.002010 (PMC11340643; doi:10.1099/jgv.0.002010)
Supplement: Uncited Fig. S1. [file jgv-105-02010-s001.pdf]

SUPPLEMENTARY FIGURES

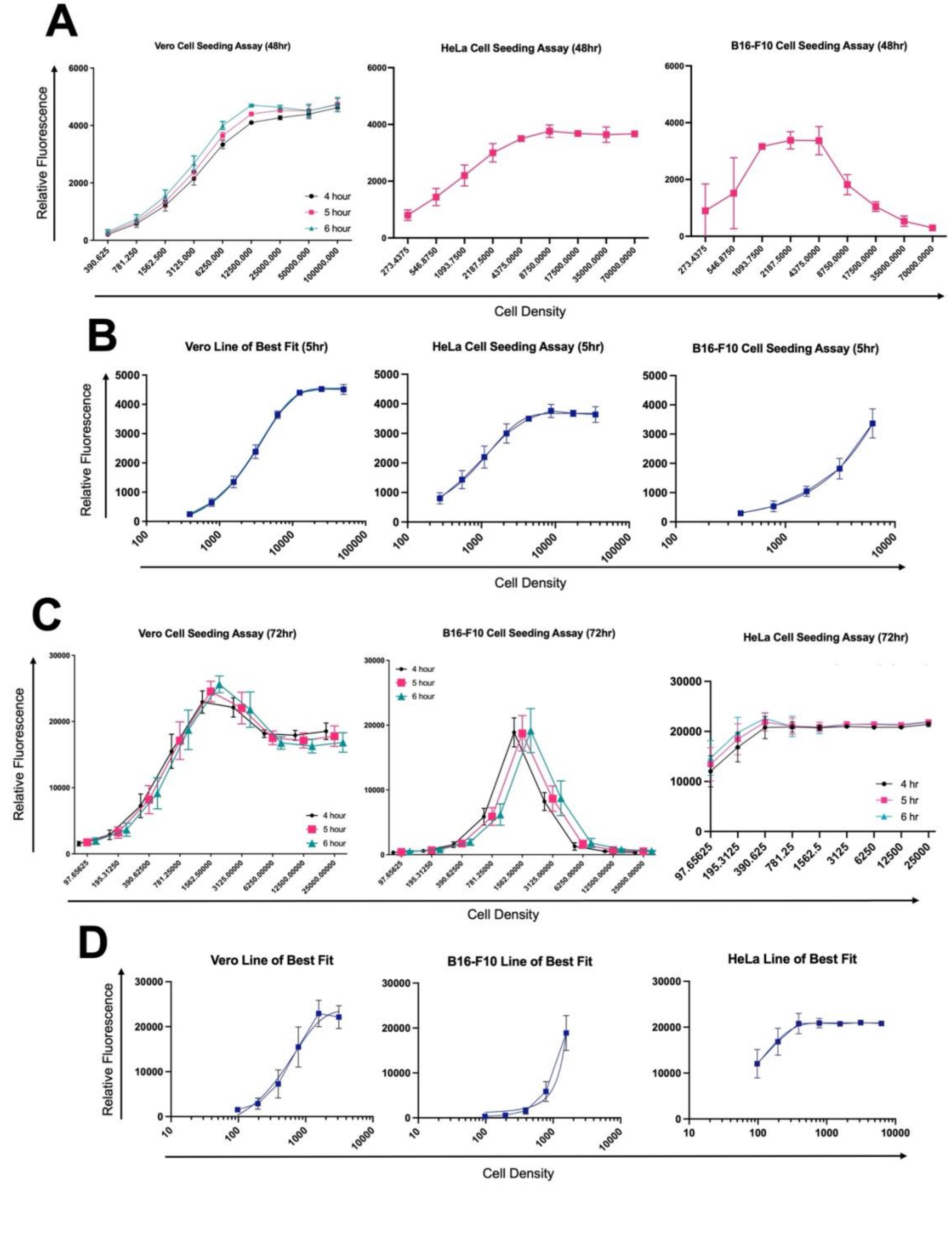

Supplementary Figure 1 *Optimization of cell seeding densities for metabolic resazurin assays.*

Graphs depicting the results of 48-hour cell seeding assays for (A) Vero, B16-F10, and HeLa cells. (B) Lines of best fit for the Vero, B16-F10, and HeLa cells five hours following addition of resazurin sodium salt solution. Graphs depicting the results of 72-hour cell seeding assays for (C) Vero and B16-F10 cells. (D) Lines of best fit for Vero and B16-F10 cells four hours following the addition of resazurin sodium salt solution. (A and C) Statistical analysis was performed using a one-way analysis of variance with Tukey's multiple comparisons to determine if there was a significant difference between four-, five-, and six-hour incubation times after the addition of resazurin sodium salt solution addition. Means and standard errors are shown. (B and D) Formulas giving the lines of best fit were used to determine the number of cells that needed to be seeded to achieve 90% confluency at the desired timepoint.

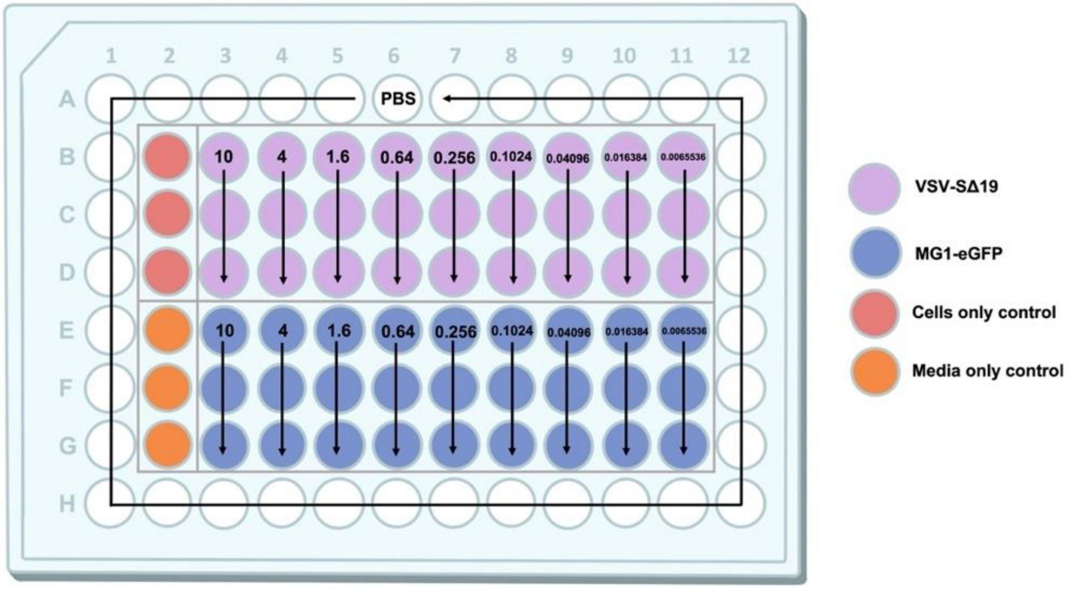

Supplementary Figure 2 *Metabolic resazurin assay 96-well plate set-up*

Schematic depicts the typical set-up of a resazurin assay 96-well plate to investigate the effect of decreased temperatures on the virus-mediated cell killing ability of MG1-eGFP and VSV-SΔ19. The perimeter of the plate is filled with phosphate buffered saline (PBS) to prevent evaporation of wells containing cells/virus. Cells only and media only wells were prepared in triplicate. Following time for cells to adhere they were treated with viruses at nine different multiplicities of infection (MOIs) in triplicate from an MOI of 10 to 0.0065536 (2.5-fold serial dilution).

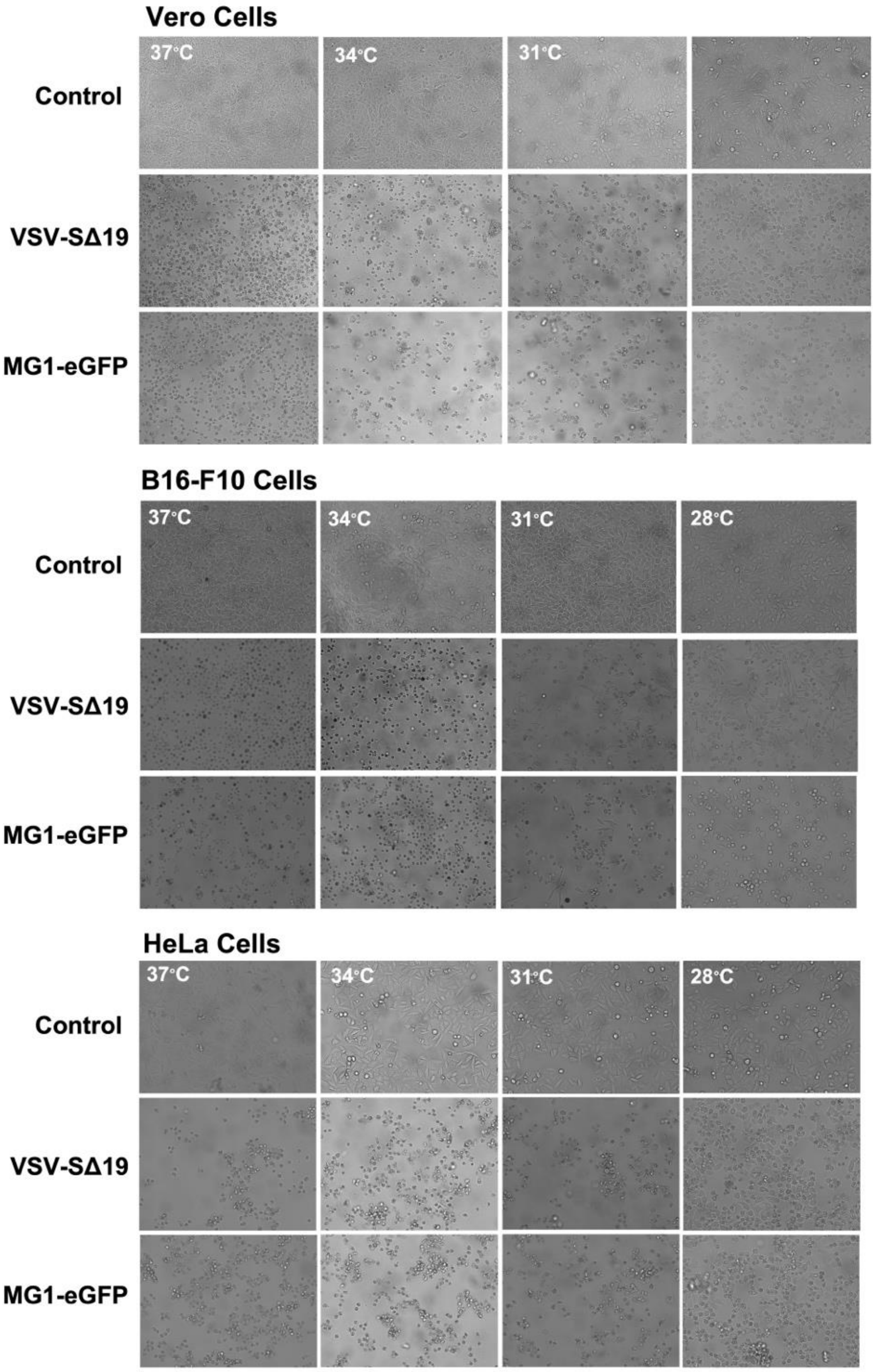
Vero Cells
37°C
34°C
31°C
Control
VSV-SΔ19
MG1-eGFP
B16-F10 Cells
37°C
34°C
31°C
28°C
Control
VSV-SΔ19
MG1-eGFP
HeLa Cells
37°C
34°C
31°C
28°C
Control
VSV-SΔ19
MG1-eGFP

Supplementary Figure 3 *The effect of decreased temperature on the virus-mediated cell killing ability of Maraba virus (MG1-eGFP) and vesicular stomatitis virus (VSV-SΔ19).*

Brightfield photos of (A) Vero, (B)B16-F10, and (C) HeLa cell cultures in 12-well plates that have been infected with VSV-SΔ19 and MG1-eGFP at an MOI of 0.1. Cells were incubated at 28.0°C, 31.0 °C, 34.0°C and 37.0°C and photos were taken 48 hours following infection.

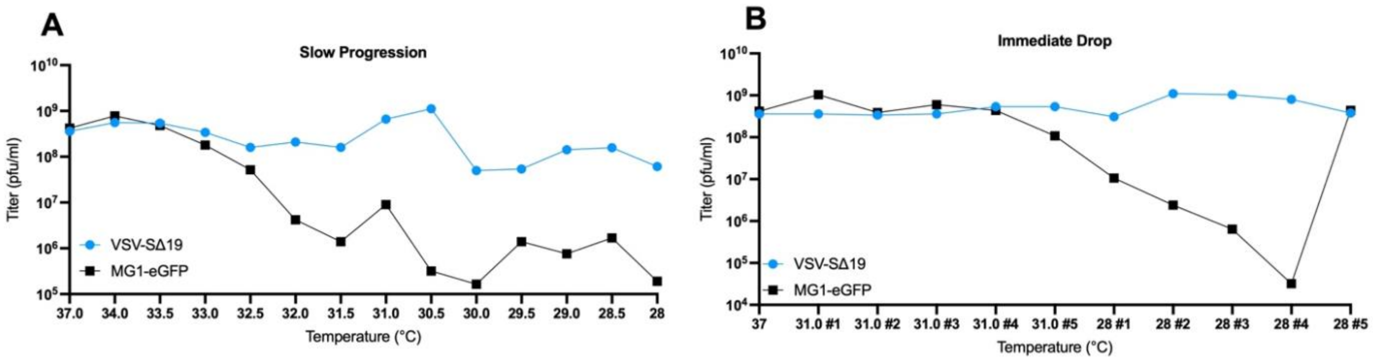

Supplementary Figure 4 *Cold-adaptation of Maraba virus (MG1-eGFP) and vesicular stomatitis virus (VSV-SΔ19).*

Graphs showing viral titers after infections of Vero cells with MG1-eGFP or VSV-SΔ19 at temperatures <37°C. (A) Vero cells were infected with MG1-eGFP or VSV-SΔ19 at a multiplicity of infection of 0.1. The initial infection was done at 37°C, the next passage was at 34°C, and the following passages were at half a degree lower each time. (B) Vero cells were infected with MG1-eGFP or VSV-SΔ19 at a multiplicity of infection of 0.1. The initial infection was at 37°C, with the subsequent five infections done at 31°C, and the final five passages done at 28°C. Viral titers (pfu/mL) were determined using a plaque assay. (n=1/treatment). MG1-eGFP was cloned by picking a colony at the second-to-last passage, in an attempt to grow up a viral clone to recover the titer before moving onto further assays.

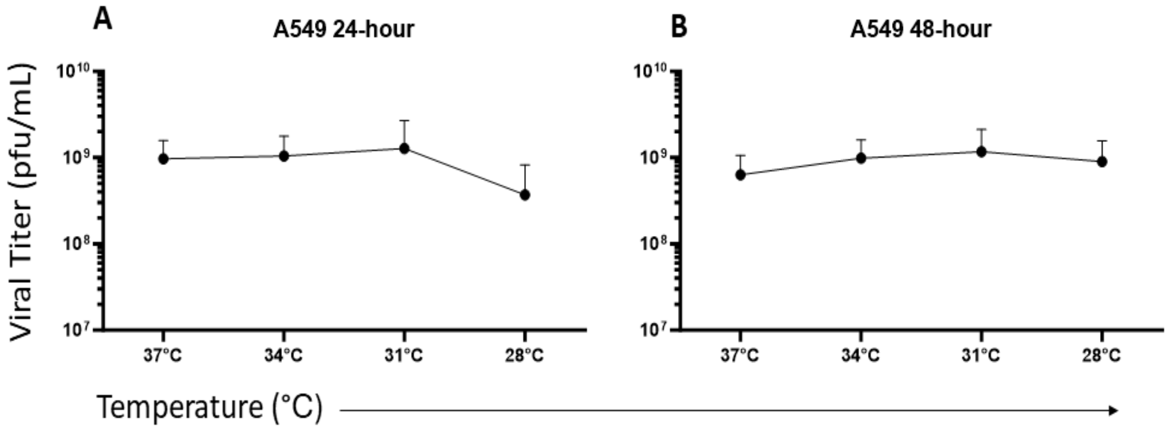

Supplementary Figure 5 *The effect of decreased temperature on the replicative ability of Vesicular Stomatitis Virus (VSV-eGFP).*

Graphs showing the results of virus yield assays conducted in A549 cells at (A) 24- and (B) 48-hours. Cells were infected at an MOI of 0.01 and incubated at 28.0°C, 31.0°C, 34.0°C and 37.0°C. Viral supernatants were collected at 24- and 48-hour timepoints and titered using the standard plaque assay and are in plaque forming units per millilitre (pfu/mL). Statistical analysis was performed using a one-way analysis of variance with Tukey's multiple comparisons. For both time points, none of the viral titers at temperatures <37°C differed from the respective titer at 37°C. Means and standard errors are shown. (n=3/treatment).

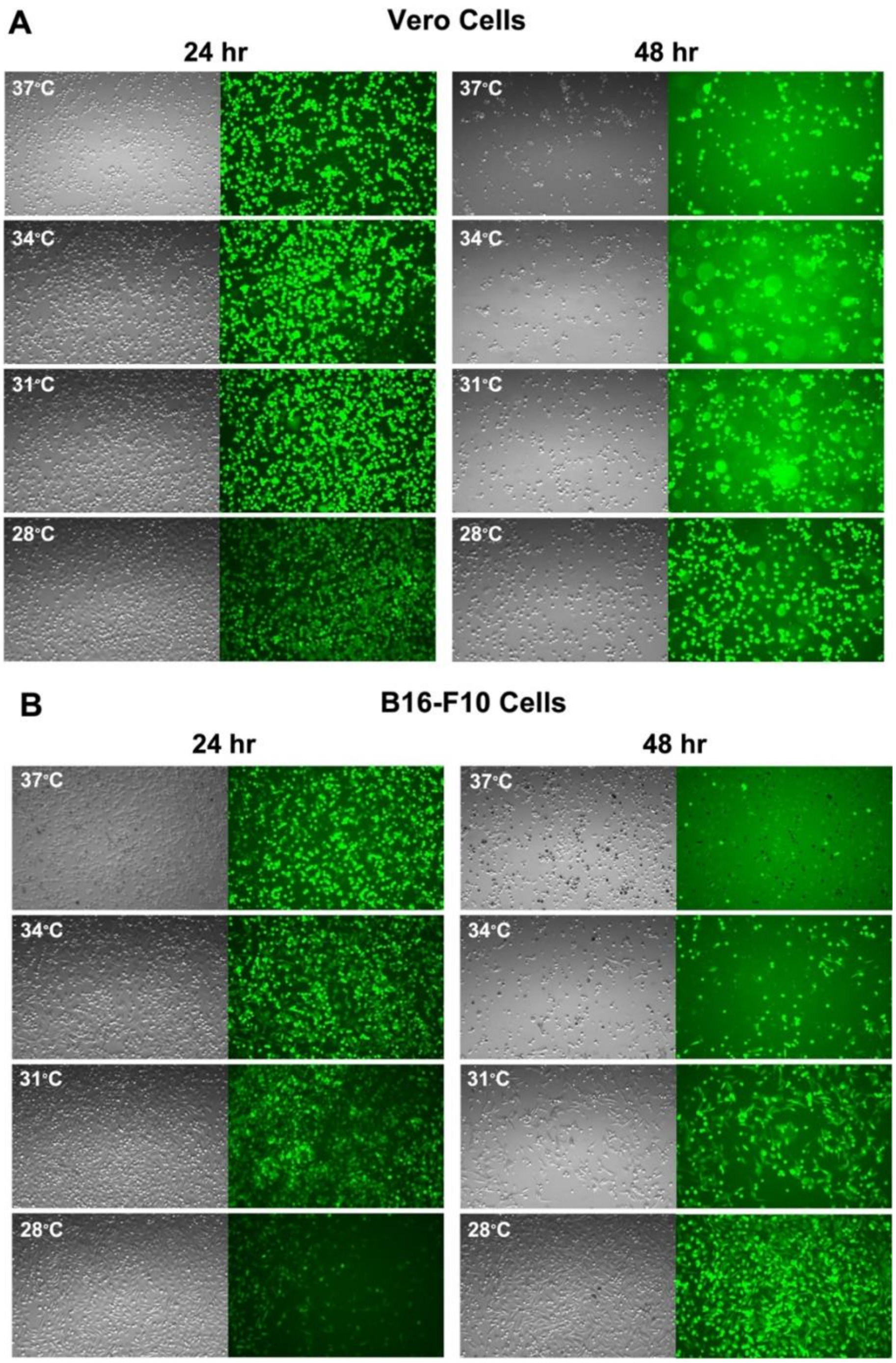

Supplementary Figure 6 *The effect of decreased temperature on the virus-mediated cell killing ability of vesicular stomatitis virus (VSV-eGFP).*

Photos taken with a microscope using brightfield and green fluorescent filters (photos on the left and right, respectively) of (A) Vero, and (B) B16-F10 cell cultures in six-well plates that were infected with VSV-eGFP at a multiplicity of infection of 0.01. Cells were incubated at 28°C, 31°C, 34°C and 37°C and photos were taken at 24- and 48-hours post-treatment.

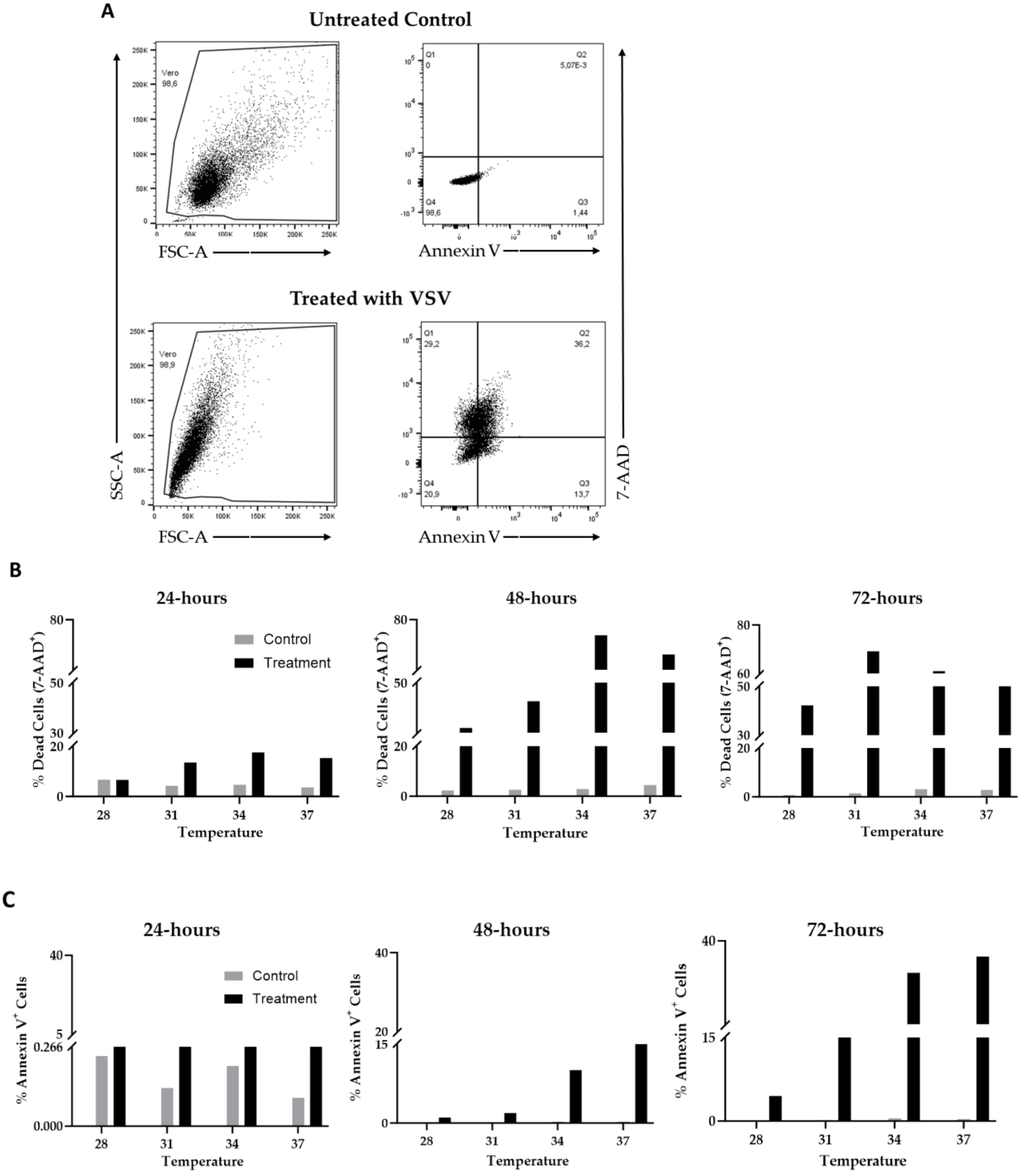
A
Untreated Control
Vero
98,6
Q1
0
Q2
5,07E-3
Q4
98,6
Q3
1,44
FSC-A
Annexin V
Treated with VSV
Vero
98,9
Q1
29,2
Q2
36,2
Q4
20,9
Q3
13,7
SSC-A
7-AAD
B
24-hours
48-hours
72-hours
Control
Treatment
% Dead Cells (7-AAD+)
Temperature
C
24-hours
48-hours
72-hours
Control
Treatment
% Annexin V+ Cells
Temperature

D

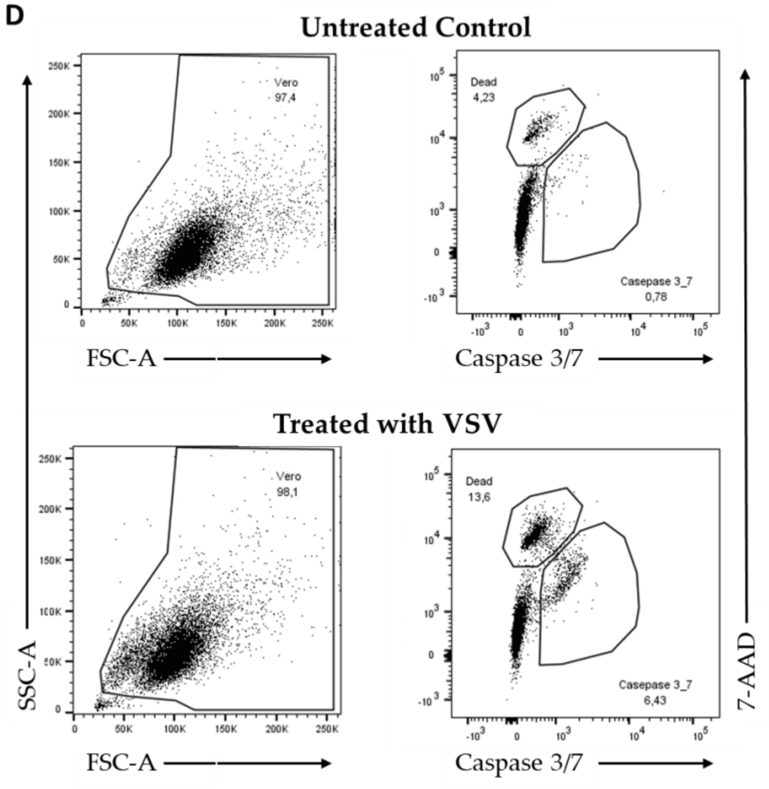

E

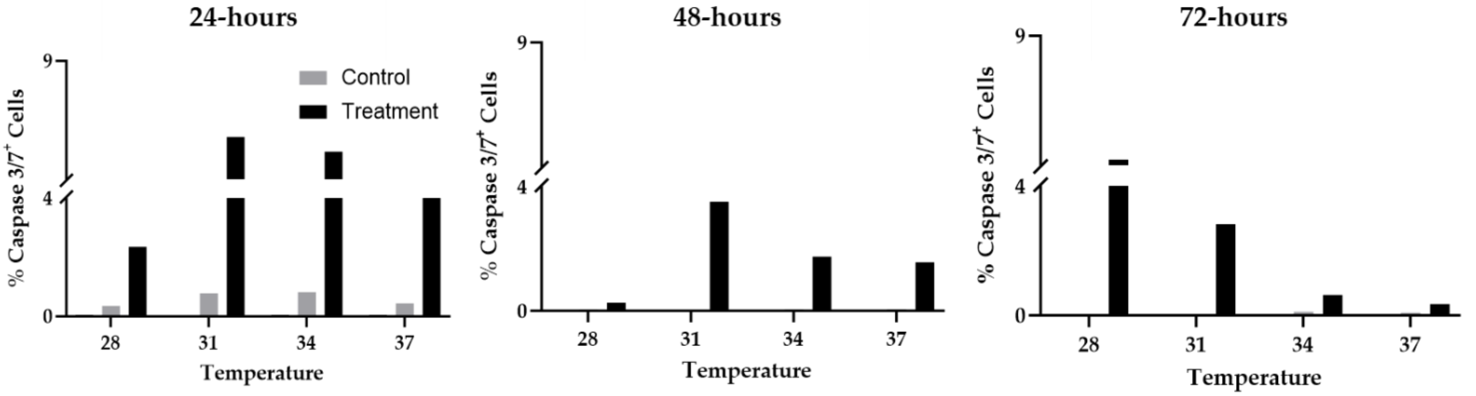

Supplementary Figure 7 *Flow cytometric assessment of the kinetics of expression of markers of cell death induced by treatment with vesicular stomatitis virus (VSV) at decreasing temperatures.*

Vero cell cultures in six-well plates were treated with VSV-eGFP at a multiplicity of infection of 0.01. Cells were incubated at 28°C, 31°C, 34°C or 37°C for 24- 48-, and 72-hours. Following treatment, cells were co-stained with 7-aminoactinomycin D (7-AAD) and annexin V. The flow cytometry gating strategy is shown in (A) and the results for 7-AAD and annexin V are shown as graphs in (B) and (C), respectively. Other cells were co-stained with 7-aminoactinomycin D (7-AAD) and anti-caspase-3/7. The flow cytometry gating strategy is shown in (D) and the results for cells expressing caspase-3/7 are shown as graphs in (E). These results are from one experimental replicate.
